# Profiles of immune cell infiltration in head and neck squamous carcinoma

**DOI:** 10.1042/BSR20192724

**Published:** 2020-02-25

**Authors:** Bin Liang, Ye Tao, Tianjiao Wang

**Affiliations:** Department of Bioinformatics, Key laboratory of Cell Biology, Ministry of Public Health, and Key Laboratory of Medical Cell Biology, Ministry of Education, School of Life Sciences, China Medical University, China

**Keywords:** Bioinformatics, Head and neck cancer, Immune cell, prognosis, TCGA

## Abstract

Tumor immune infiltration cells (TIICs) are highly heterogeneous, not only in different cancer subtypes but also within different cancer regions. We conducted the Cell-type Identification using Estimating Relative Subsets Of RNA Transcripts (CIBERSORT) method. We assessed the relative proportions of 22 TIICs in HNSC using publicly available TCGA transcriptional datasets, analyzed the proportions of TIICs between HNSC tissues and normal tissues, along with accompanying clinicopathological data, and the impact of TIICs on clinical outcome. After the filter criteria, a total of 395 patients were included in the analysis. We found significant differences in naïve B cells, monocytes, resting mast cells, activated mast cells, CD8^+^ T cells, and M0 macrophages between HNSC tissues and adjacent non-cancer tissues. We also found that some TIIC subgroups were significantly associated with clinical parameters. Moreover, the patients with low Tregs fraction had worse OS and DFS than those with high Tregs fraction. However, low M0 macrophages fraction was associated with better OS and DFS in HNSC patients. Moreover, Tregs and M0 macrophages are likely to be important determinants of prognosis, which may serve as a potential immunotherapy target for HNSC. Then, we screened the immune-related differentially expressed genes (DEGs), performed the GO and KEGG enrichment analysis, constructed the protein–protein interaction network, and screened the prognosis-related hub genes in HNSC. However, further clinical investigation and basic experiments are needed to validate our results, and uncover the molecular mechanisms interlinking TIICs in HNSC and their roles in prognosis and therapy.

## Introduction

Head and neck cancer (HNC) is the sixth most common malignancy worldwide, with an incidence rate of approximately 600,000 patients per year [1]. Approximately 95% of HNC cases are head and neck squamous carcinoma (HNSC), commonly arising from the mucosa of oral cavity, oropharynx, hypopharynx, and larynx [2,3]. The epidemiology surveys showed that smoking and alcohol consumption, and human papillomavirus (HPV) infection are most common causative factors for HNSC [4–6]. Although there have been certain advances in HNSC treatment, in stage III and IV HNSC, 5-year survival rate is less than 50% [7]. Despite significant advances in multidisciplinary treatments, including monoclonal antibody therapies, adoptive T-cell transfer, cancer vaccines, and cytokine therapy, the overall survival rate has remained stable for decades [8]. At present, it seems increasingly clear that HNSC are characterized by a large heterogeneity in terms of potential risk factors, complex molecular abnormalities, and varied tumor sites.

Over the past decade, investigations have increasingly focused on the tumor microenvironment (TME). TME is a complex ecosystem and an active participant in all stages of cancer initiation and progression, which consists of a complex system of immune cells, including subsets of T cells, B cells, dendritic cells, macrophages, and NK cells [9,10]. First, due to the abundance of lymph nodes and vessels in head and neck, activation of local immunity may play a role in limiting the spread of HNSC and/or enhancing the HNSC patient response to therapy [11]. Second, the presence of tumor immune infiltration cells (TIICs) in cancer tissues were significantly correlated with alcohol drinking and smoking experience, implying a linkage between inflammatory processes affecting the smokers or alcohol drinkers and immune response status [12]. Notably, tobacco and alcohol are two main and classical risk factors for HNSC [13]. Third, most HNSCs are resistant to immunotherapy, due to deficient immune surveillance and the presence of immunosuppressive mediators in TME [14]. The early studies have generated strong interest in the investigation of TIICs in TME in HNSC. Schneider et al. reported that HNSC with high p16 expression showed elevated T- and B-lymphocyte infiltration and a favorable prognosis, which suggests a prognostic relevance of immune cell infiltration [15]. Recent study indicated that subtypes with enhanced immune microenvironment in HNSC were associated with immune features, immune checkpoint molecules, and patient outcomes, providing new light on the strategy of immunotherapy in HNSC [16]. Therefore, understanding the role of TIICs in HNSC is potential in predicting response to therapy and in directing appropriate therapies.

At present, many researchers aimed to explore the molecular mechanism from bulk gene expression data in tumors using bioinformatics approach, which provided an efficient method for systematically screening tumor-related genes and identifying various immune regulatory mechanism in individual therapy [17]. Saloura et al. identified the presence or absence of CD8+ T-cell infiltration influenced the TME, varied signaling pathways and gene alteration in HNSC by analyzing the TCGA data [18]. Chen et al. identified two microenvironment-based subtypes by mining TCGA data, including active immune response and exhausted immune response, and different subtypes were characterized by various immune cell infiltration, signaling pathways and clinical outcomes [19]. TIICs are highly heterogeneous, not only in different cancer subtypes but also within different cancer regions, even patients with the same cancer types. In the present study, we conducted the Cell-type Identification By Estimating Relative Subsets Of RNA Transcripts (CIBERSORT) method, which is superior to other methods [20]. Next, we assessed the relative proportions of 22 TIICs using publicly available TCGA transcriptional datasets, presenting a comprehensive immune cell landscape of HNSC. Then, we analyzed the proportions of TIICs between HNSCC tissues and normal tissues, along with accompanying clinical pathological data, in order to illuminate the TIICs landscape of HNSC, and indicate the correlation between TIICs and clinical factors, such as TNM stage, grade, and smoking and drinking habit, and impact of TIICs on clinical outcome. Furthermore, we analyzed the immune-related differentially expressed gene (DEGs), performed the functional enrichment analyses, constructed the protein–protein interaction network, and evaluated the prognostic values of hub genes in HNSC patients.

## Materials and methods

### Datasets

All level 3 RNA expression data from 529 HNSC cancer tissues and 57 adjacent-cancer tissues were obtained from The Cancer Genome Atlas (TCGA) database ((https://tcga-data.nci.nih.gov/tcga/) for the purposes of the present study. The clinical information of HNSC patients, including gender, diagnosis age, clinical stage, T stage, lymph node involvement, pathological grade, life habit, survival status, and survival duration in months were downloaded from from cBioPortal for Cancer Genomics (http://www.cbioportal.org/). According to the publication guidelines, datasets may be used for publication without restriction or limitation.

### Immune infiltration analysis based on CIBERSORT method

CIBERSORT is a useful tool for high-throughput characterization of TIICs, from complex tissues based on RNA sequencing data. A validated leukocyte gene signature matrix (LM22) was used to identify the 22 functionally defined immune cell subtypes. These immune cell components included naïve B cells, memory B cells, plasma cells, 7 T cell types (CD8^+^ T cells, naïve CD4^+^ T cells, resting CD4^+^ memory T cells, activated CD4^+^ memory T cells, follicular helper T cells, Tregs, γδ T cells), macrophages (M0 macrophages, M1 macrophages, M2 macrophages), resting mast cells, activated mast cells, resting NK cells, activated NK cells, resting dendritic cells (resting DC), activated dendritic cells (activated DC), monocytes, eosinophils, and neutrophils. The filter criteria of each sample is set as the CIBERSORT calculation of *P*  <  0.05, indicating that the inferred proportion of each TIICs subtype are accurate and suitable for further analysis. In each sample, the proportion of all TIICs equal to 1.

### Identification of differentially expressed genes

The “limma” package in R was applied to screen the differentially expressed genes (DEGs) between HNSC samples and adjacent non-cancer tissues. The thresholds of DEGs were as follows: log_2_|fold change (FC)| >1, *P* < 0.05, and false discovery rate (FDR) < 0.05. Venn diagram was used to analyze the overlapping genes between DEGs and a validated leukocyte gene signature matrix in CIBERSORT.

### Function enrichment analysis

To further understand the function of overlapping genes, Gene Ontology (GO) annotation and Kyoto Encyclopedia of Genes and Genomes pathway (KEGG) analyses of common DEGs were analyzed by the Database for Annotation, Visualization, and Integrated Discovery database (DAVID, version 6.8, http://david.ncifcrf.gov). GO consists of biological processes, cell components, and molecular processes. *P* < 0.05 was considered to be significant.

### Construction of protein–protein interaction network

The protein–protein interaction (PPI) network of common genes was constructed using The Search Tool for the Retrieval of Interacting Genes (STRING) database (version 11.0, https://string-db.org/). The minimum required interaction score was set as 0.4. The PPI network was visualized with Cytoscape software (version 3.7.1, https://cytoscape.org/). The “CytoHubba” plug-in was used to identify the hub genes in the PPI network. The “Molecular Complex Detection” (MCODE) plug-in was applied to screen the key modules of the PPI network. The GO and KEGG pathway analyses were performed to analyze the key modules.

### Prognosis analysis of hub genes

The prognosis of the top 20 hub genes was evaluated by GEPIA database (http://gepia.cancer-pku.cn/). GEPIA is an interactive web application for gene expression analysis based on 9736 tumors and 8587 normal samples from the TCGA and the Genotype-Tissue Expression (GTEx) databases [21]. *P* < 0.05 was considered statistically significant.

### Statistical analysis

All statistical analyses were performed in SPSS 20.0 statistical software (SPSS, Chicago, IL), R v3.3.2 and Bioconductor software package (https://www.bioconductor.org/). The different proportions of TIICs between HNSC tissues and adjacent non-cancer tissues were compared by Student’s *t* test. We evaluated the relationships between each TIIC proportion and clinicopathological characteristics in HNSC patients using one-way analysis of variance (ANOVA). Overall survival (OS) and disease-free survival (DFS) curves was calculated by Kaplan–Meier method and tested by log-rank test. The univariate and multivariate Cox proportional hazards regression models were conducted to examine the prognostic value of TIICs and clinicopathological parameters in HNSC. *P* < 0.05 was considered statistically significant.

## Results

### Patient characteristics

The TCGA database included 529 HNSC samples. After the filter criteria: CIBERSORT calculations of *P* < 0.05, a total of 395 patients were included in the analysis. The clinicopathological characteristics are shown in Table 1. The median age at diagnosis was 61.3 ± 12.1 years (range 19.0–88.0 years) and 280 (70.9%) of the patients were males.

**Table 1:** Clinical characteristics of included HNSC patients after applying data filter criteria

Characteristics	Number of patients	Percentage (%)
Number of patients	395	
Age at diagnosis (years)		
≤60	184	46.6%
>60	211	53.4%
Gender		
Male	280	70.9%
Female	115	29.1%
Clinical stage		
Stage I+II	91	23.0%
Stage III+IV	304	77.0%
Metastasis		
M0	390	98.7%
M1	5	1.3%
N stage		
N0	198	50.1%
N2	197	49.9%
T stage		
T1+T2	143	36.2%
T3+T4	252	63.8%
Grade		
G1+G2	287	72.7%
G3+G4	97	24.5%
Gx	11	2.8%
Alcohol history		
No	120	30.4%
Yes	266	67.3%
NA	9	2.3%
Smoking history		
1+2	217	54.9%
3+4	170	43.0%
NA	8	2.1%

### The distribution of TIICs in HNSC

**Figure 1:**
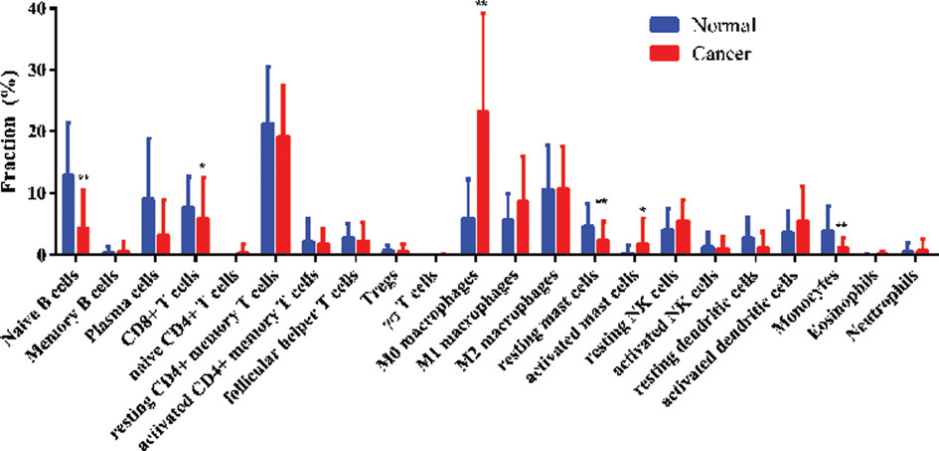
Comparison of 22 TIICs between HNSC tissues and adjacent non-cancer tissues *P<0.05; **P<0.01.

**Figure 2:**
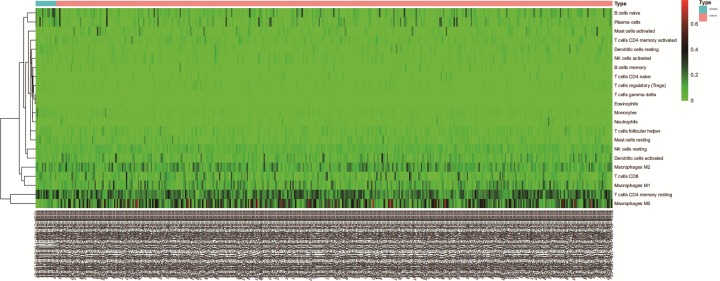
Relative TIICs proportions evaluated in HNSC samples by CIBERSORT

**Figure 3:**
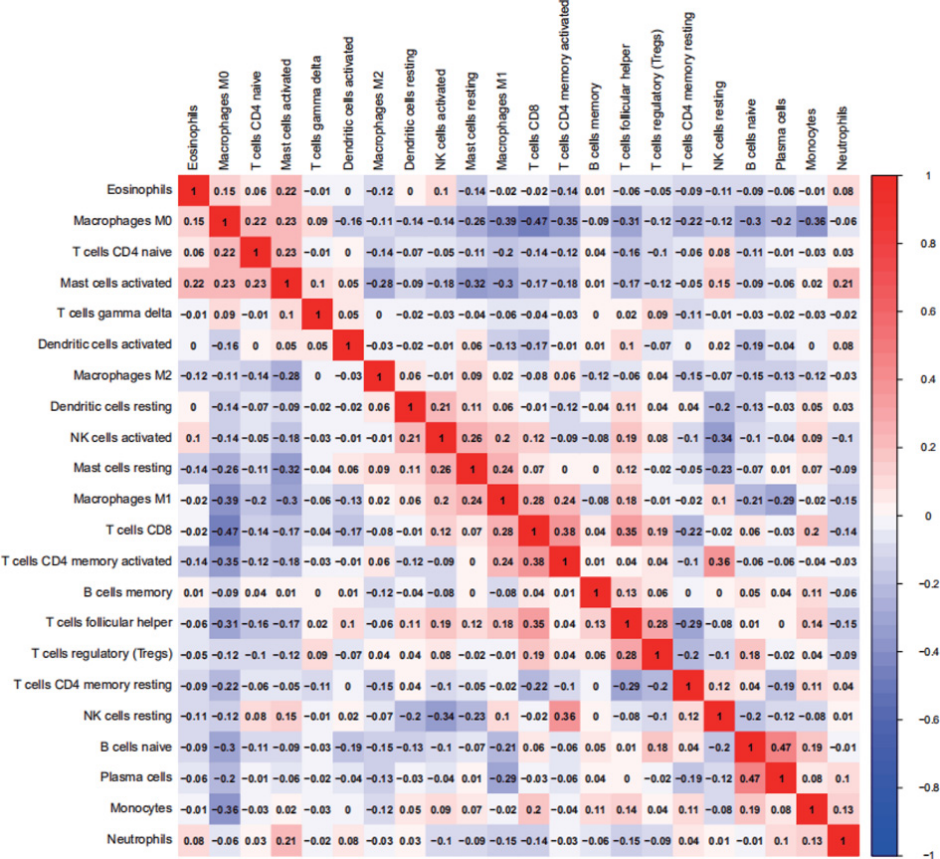
Correlation matrix of 22 immune cell proportions in HNSC samples

The composition of TIICs in HNSC tissues and adjacent non-cancer tissues was compared. The proportions of naïve B cells (*P* < 0.001), monocytes (*P* < 0.001), resting mast cells (*P* = 0.005), and CD8^+^ T cells (*P* = 0.043) in HNSC tissues were significantly lower than adjacent non-cancer tissues, while the proportion of activated mast cells (*P* = 0.025) and M0 macrophages (*P* < 0.001) in HNSC tissues was significantly higher than adjacent non-cancer tissues (Figure 1). The percentages of 22 TIICs in HNSC and adjacent non-cancer tissues were shown using heatmap (Figure 2). The relative percent of each TIIC in HNSC sample were shown in Supplementary Figure S1. The correlation of 22 TIICs were calculated (Figure 3). The CD8^+^ T cells was significantly positively correlated with activated CD4^+^ memory T cells (*r* = 0.38, *P* < 0.001), but was significantly negatively correlated with M0 macrophages (*r* = −0.47, *P* < 0.001).

### Association between TIICs and clinicopathological parameters in HNSC patients

**Figure 4:**
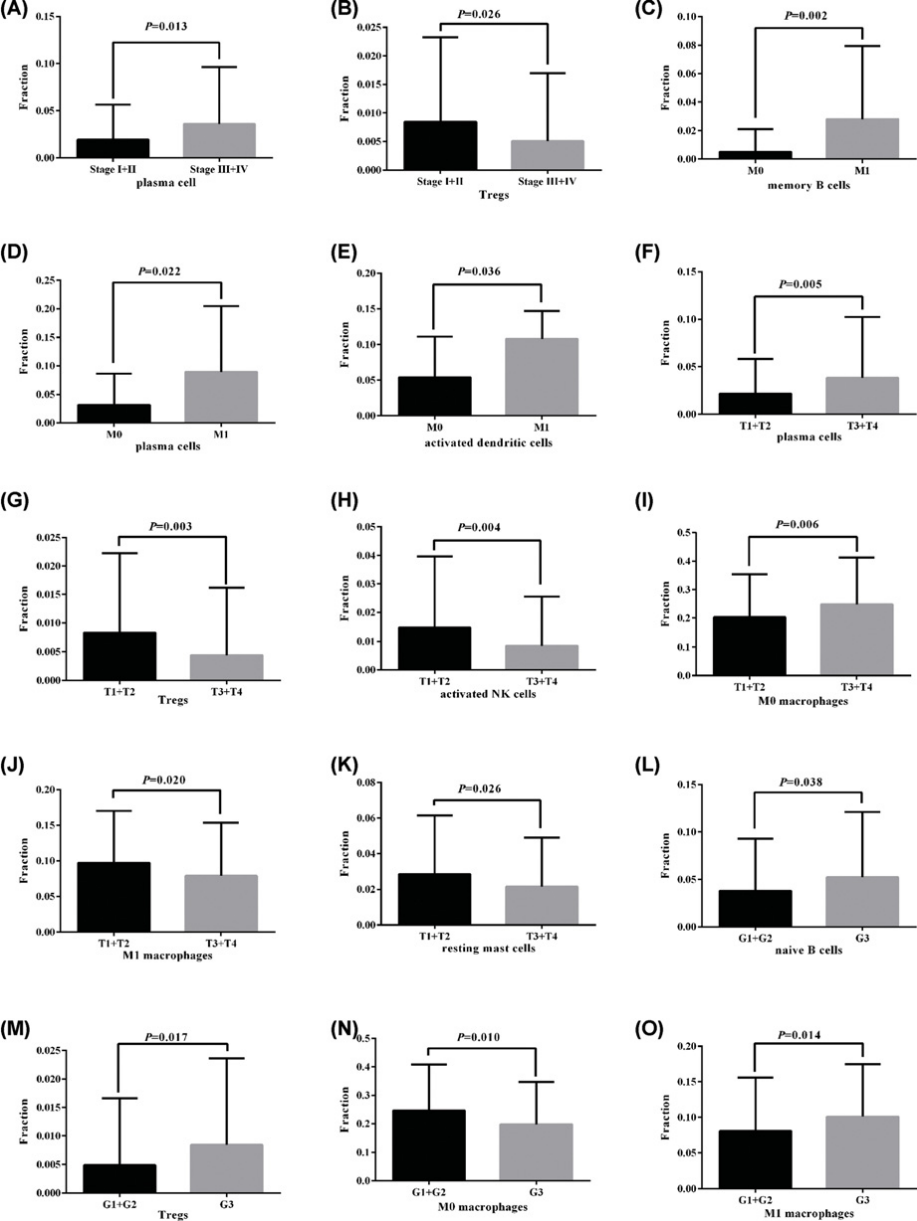
Association between TIICs and clinical parameters (**A** and **B**) Plasma cells fraction and Tregs were significantly associated with clinical stage. (**C–E**) Memory B cells, plasma cells, and activated dendrite cells fractions were significantly associated with cancer distant metastasis. (**F–K**) Plasma cells, M0 macrophages, Tregs, activated NK cells, M1 macrophages, and resting mast cells were significantly associated with T stage. (**L–O**) Naïve B cells, Tregs, M0 macrophages, and M1 macrophages were significantly associated with pathological grade.

High plasma cells fraction was significantly associated with advanced clinical stage (*P* = 0.013), but low Tregs were significantly associated with advanced clinical stage (*P* = 0.026) (Figure 4A,B). High memory B cells (*P* = 0.002), plasma cells (*P* = 0.022), and activated dendrite cells (*P* = 0.036) fractions were significantly associated with cancer distant metastasis (Figure 4C–E). High plasma cells (*P* = 0.005) and M0 macrophages (*P* = 0.006) fractions were significantly higher in advancer T stage (T3/T4), but Tregs (*P* = 0.003), activated NK cells (*P* = 0.004), M1 macrophages (*P* = 0.020), and resting mast cells (*P* = 0.026) were significantly lower in advancer T stage (T3/T4) (Figure 4F–K). Moreover, high naïve B cells (*P* = 0.038), Tregs (*P* = 0.017), and M1 macrophages (*P* = 0.014) were significantly associated with advancer pathological grade (G3), but low M0 macrophages (*P* = 0.010) was significantly associated with advancer pathological grade (G3) (Figure 4L–O).

### Association of TIICs proportions with prognosis in HNSC patients

**Figure 5:**
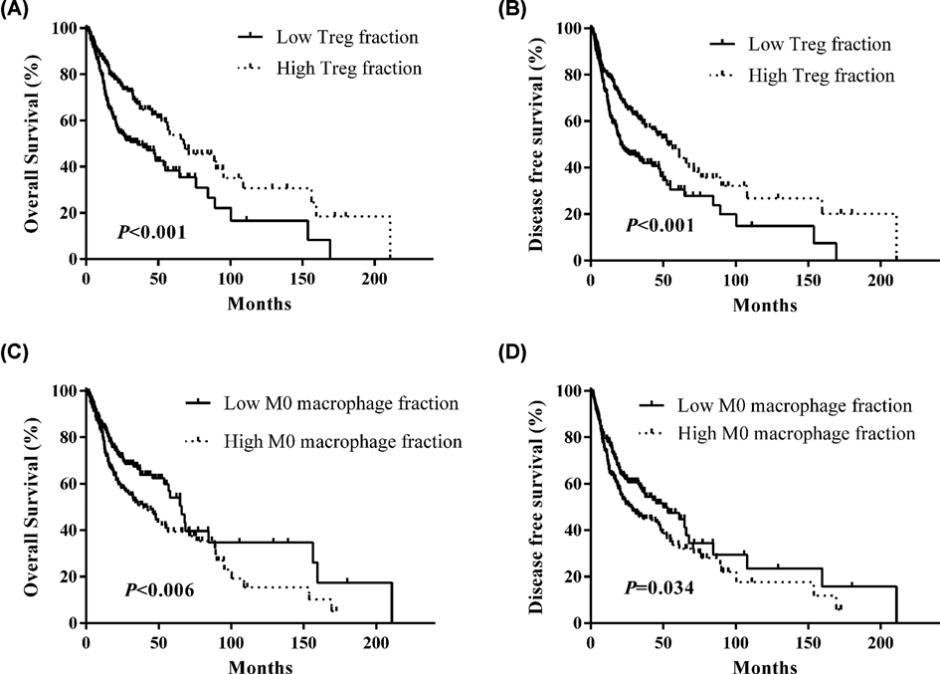
Survival plots of Treg and M0 macrophages stratified by median (**A** and **B**) Low Tregs fraction had worse OS and DFS in HNSC patients. (**C** and **D**) Low M0 macrophages fraction was associated with better OS and DFS in HNSC patients.

We further investigated the clinical prognosis of TIICs in HNSC patients. Patients with low Tregs fraction had worse OS (Figure 5A) and DFS (Figure 5B) than those with high Tregs fraction. However, low M0 macrophages fraction was associated with better OS and DFS in HNSC patients, whereas high M0 macrophages fraction predicted poor outcome in HNSC patients (Figure 5C,D). Moreover, a univariate and multivariate Cox proportional hazard model was performed to identify the independent prognostic factors for HNSC. The results showed that Tregs and M0 macrophages could serve as independent prognostic parameters for OS [Treg, HR: 0.664 (0.481–0.915), *P* = 0.012; M0 macrophages, HR: 1.543 (1.121–2.125), *P* = 0.008] and DFS [Treg, HR: 0.659 (0.490–0.870), *P* = 0.004; M0 macrophages, HR: 1.357 (1.014–1.814), *P* = 0.040] in HNSC patients, as shown in Table 2. Concurrently, distant metastasis was also an independent prognostic factor for HNSC [OS, HR: 8.836 (3.171–24.626), *P* < 0.001; DFS, HR: 5.151 (1.896–13.994), *P* = 0.001].

**Table 2:** Cox proportional-hazard regression analysis for overall survival and disease-free survival in HNSC patients

Variables	Univariate analysis			Multivariate analysis		
	HR	95% CI	*P*	HR	95% CI	*P*
Overall survival						
Age (> 60 years vs. ≤ 60)	1.174	0.868–1.587	0.297			
Gender (male vs. female)	0.694	0.509–0.948	0.021			
Stage (III+ IV vs. I+II)	1.219	0.844–1.759	0.291			
Metastasis (M1 vs. M0)	4.695	1.723–12.796	0.003	8.836	3.171–24.626	<0.001
N stage (N1-3 vs. N0)	1.219	0.905–1.642	0.192			
T stage (T3+T4 vs. T1+T2)	1.379	0.994–1.915	0.055			
Grade (G3+G4 vs. G1+G2)	0.926	0.655–1.309	0.663			
Alcohol (Yes vs. No)	0.933	0.676–1.287	0.671			
Smoking (3+4 vs. 1+2)	0.772	0.565–1.056	0.105			
Treg (high fraction vs. low fraction)	0.560	0.413–0.759	<0.001	0.664	0.481–0.915	0.012
Macrophage M0 (high fraction vs. low fraction)	1.531	1.129–2.077	0.006	1.543	1.121–2.125	0.008
Disease free survival						
Age (>60 years vs. ≤60)	1.150	0.870–1.520	0.326			
Gender (male vs. female)	0.752	0.563–1.005	0.054			
Stage (III+ IV vs. I+II)	1.229	0.875–1.725	0.234			
Metastasis (M1 vs. M0)	3.443	1.270–9.332	0.015	5.151	1.896–13.994	0.001
N stage (N1-3 vs. N0)	1.136	0.862–1.496	0.365			
T stage (T3+T4 vs. T1+T2)	1.343	0.996–1.813	0.053			
Grade (G3+G4 vs. G1+G2)	0.846	0.609–1.177	0.321			
Alcohol (yes vs. no)	1.089	0.804–1.473	0.583			
Smoking (3+4 vs. 1+2)	0.824	0.619–1.097	0.185			
Treg (high fraction vs. low fraction)	0.606	0.458–0.802	<0.001	0.659	0.490–0.870	0.004
Macrophage M0 (high fraction vs. low fraction)	1.350	1.022–1.784	0.035	1.357	1.014–1.814	0.040

Note: OS, overall survival; DFS, disease-free survival; HR, hazard ratio; CI, confidence interval.

### Identification of common DEGs

**Figure 6:**
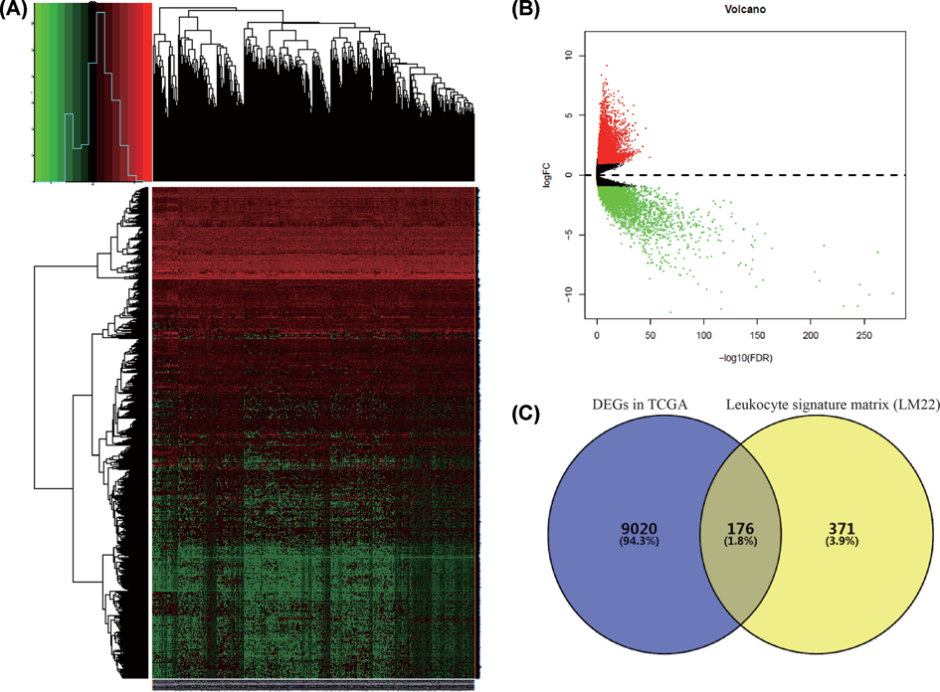
Overlapping differentially expressed genes between HNSC tissues and adjacent non-cancer tissues (**A**) Heatmap of DEGs. (**B**) Volcano plot of DEGs. (**C**) Overlapping DEGs between DEGs in TCGA database and leukocyte signature gene matrix (LM22).

Base on the threshold, log_2_|fold change (FC)| >1, *P* < 0.05, and FDR < 0.05, a total of 9196 DEGs were identified, including 5739 up-regulated genes and 3437 down-regulated genes, as shown in heatmap and volcano plot (Figure 6A,B). A validated leukocyte gene signature matrix in CIBERSORT includes 547 genes, which are used to evaluate the fraction of 22 TIICs. Venn plot identified 176 common genes between DEGs in TCGA database and validated leukocyte gene signature matrix in CIBERSORT (Figure 6C).

### GO and KEGG enrichment analyses

**Figure 7:**
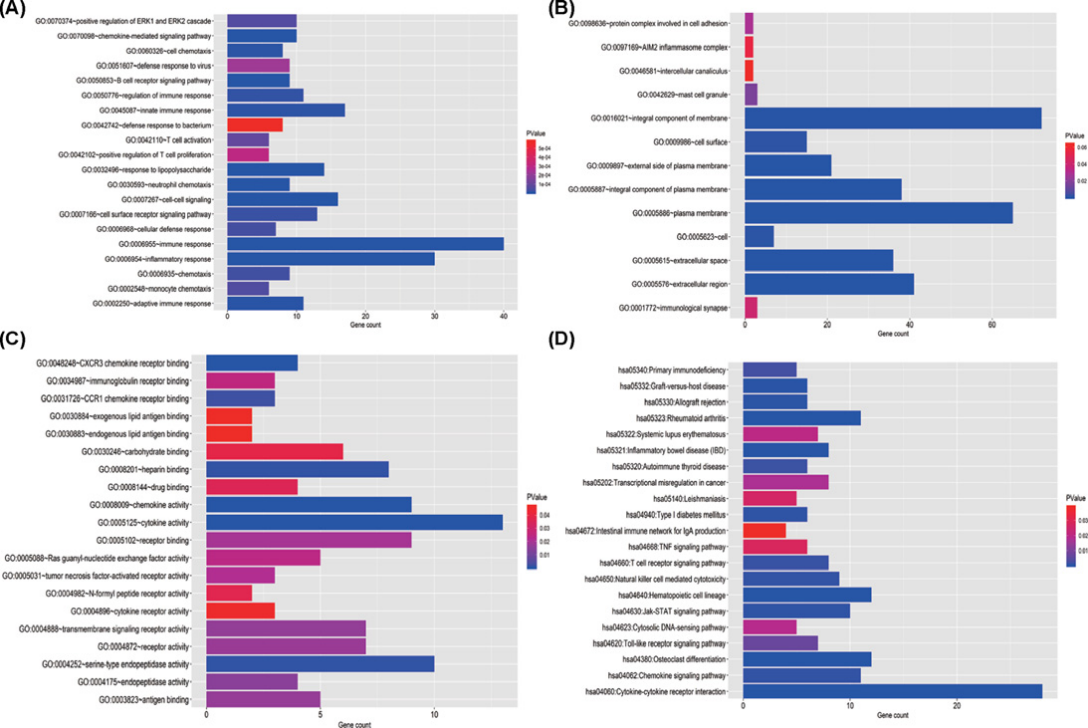
Function enrichment analysis of common DEGs (**A**) Biological process. (**B**) Cell component. (**C**) Molecular function. (**D**) KEGG pathway.

The GO and KEGG enrichment analyses were performed for the 176 common genes. The biological processes mainly involved immune response, inflammatory response, and cell–cell signaling (Figure 7A). The common DEGs were mainly located in the integral component of membrane, plasma membrane and extracellular region (Figure 7B). The molecular function of common DEGs included cytokine activity, serine-type endopeptidase activity, and chemokine activity (Figure 7C). The main KEGG pathway of common DEGs mainly involved in cytokine–cytokine receptor interaction, hematopoietic cell lineage, and rheumatoid arthritis (Figure 7D).

### Construction of PPI network

**Figure 8:**
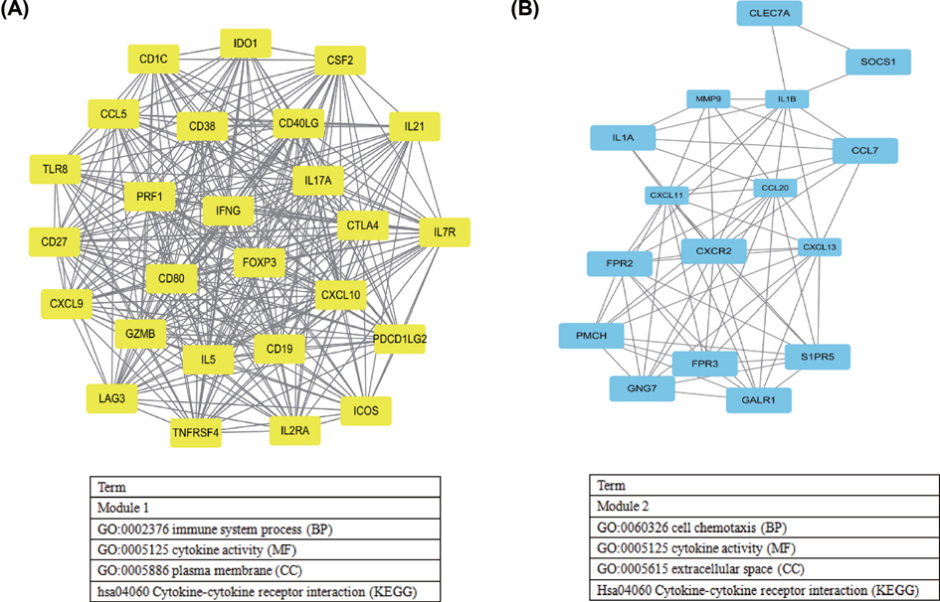
Two key modules of protein–protein interaction network and related functions (**A**) Module 1 (score: 23.52), (**B**) Module 2 (score: 9.33).

The PPI network of 176 common DEGs, which was originated from STRING database, was constructed to predict the interactions of common DEGs, consisting of 170 nodes and 877 edges (Supplementary Figure S1). The top 20 hub genes were screened using the CytoHubba plug-in, and included CXCL10, CCL5, IL17A, TLR8, CSF2, IL1B, CXCL9, IFNG, CTLA4, IL5, FOXP3, CD40LG, CD80, GZMB, CD19, MMP9, PRF1, CCL20, CD27, and CXCL11. In addition, we conducted the MCODE analyses to identify the two key modules in the PPI network (Figure 8A,B). The main GO terms and KEGG pathway analyses of DEGs in two modules were mainly involved in immune response, cell chemotaxis, and cytokine–cytokine receptor interaction (Figure 8).

### Prognostic analysis of hub genes

**Figure 9:**
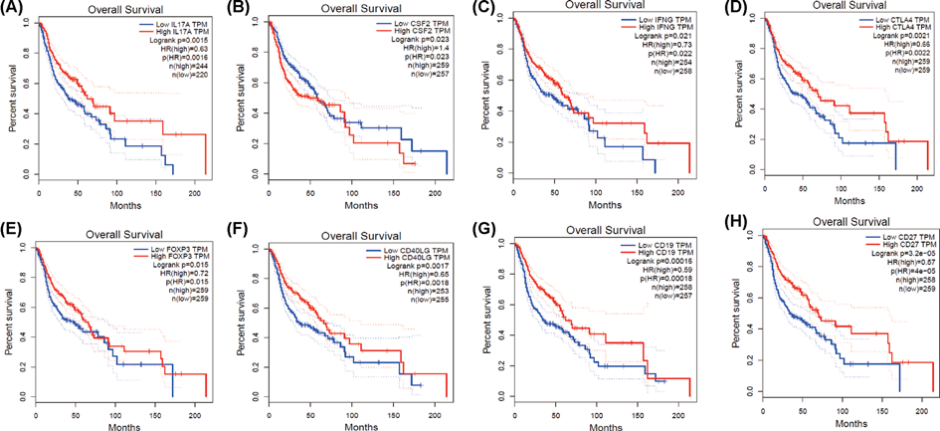
Kaplan–Meier plots of hub genes by GEPIA database (**A**) IL17A, (**B**) CSF2, (**C**) IFNG, (**D**) CTLA4, (**E**) FOXP3, (**F**) CD40LG, (**G**) CD19, and (**H**) CD27.

Kaplan–Meier curve analysis was performed to evaluate the OS for the top 20 hub genes using GEPIA database. The analyses indicated that high IL17A, IFNG, CTLA4, FOXP3, CD40LG, CD19, and CD27 levels were significantly associated with favorable overall survival, whereas low CSF2 predicted a better overall survival (all *P* < 0.05, Figure 9).

## Discussion

Immune cells in TME may exert either tumor-promoting or tumor-suppressive effects [22]. Accumulating evidence has demonstrated that HNSC cells have the ability to organize an immunosuppressive microenvironment to evade immune recognition, which not only involved in the development and progression of HNSC, but also determined the clinical course of HNSC. In the present study, we used an analytical strategy to provide comprehensive view of 22 TIICs, including B cell, T cell, macrophages, mast cells, NK cells, dendritic cells, monocytes, eosinophils, and neutrophils. Then, we evaluated the proportions of TIICs in solid HNSC tissues, and found the differences of TIICs between cancer tissues and adjacent non-cancer tissues. Moreover, we identified some TIICs were associated with clinicopathological parameters, and determined that Tregs and M0 macrophages were independent prognostic factors in HNSC patients.

The TIICs mainly includes B cells, T cells (CD8^+^ T cells, CD4^+^ T cells, follicular helper T cells, and Tregs), macrophages, and NK cells, which play key roles in tumor promotion and/or tumor suppression [23,24]. B cells, as precursors of antibody-producing plasma cells, are an important component of the adaptive immune system, and could interact with other immune cells through cytokine secretion and antigen presentation [25]. The specific signals drive the differentiation of human naive B cells into memory B cells and plasma cells, all of which play distinct roles during humoral immune responses [26]. T cells play critical roles in tumor control [27], but some T cells can also promote the progression of tumors through different growth factors. Accumulating evidence has demonstrated that the presence of tumor specific T cells has been correlated with improved clinical outcome in different human cancers [28,29], such as CD8^+^ T cell and CD4^+^ T cell [30,31], whereas regulatory T cells (Tregs) could accumulate aberrantly in some types of tumor to suppress antitumor immunity and support the establishment of immunological hypo-responsiveness microenvironment [32]. Tumor-associated macrophages (TAMs) are an important component among tumor infiltrating immune cells, and are also key regulators between inflammation and cancer [33,34]. Based on two distinctly different polarizations, macrophages are divided into two categories: classical M1 and alternative M2 macrophages, which function both anti- and pro-tumor effects, respectively [35]. TAMs play a controversial role in tumor progression depending on different tumor types. TAMs infiltration has been shown to correlate with worse outcome in cancers, including breast cancer [36], oesophageal cancer [37], gastric cancer [38], and pancreatic cancer [39]. However, some studies indicated that TAMs were correlated with better prognosis in non-small cell lung cancer [40] and colorectal carcinoma [41]. Mast cells (MCs), a type of innate immune cells, are a potent proangiogenic factor in solid tumors, in which mast cell accumulation correlated with increased neovascularization, mast cell VEGF expression, tumor aggressiveness, and poor prognosis [42]. The present studies have revealed that mast cells have a pro-tumor or anti-tumor role depending on the type of cancer, the degree of tumor progression, and the location of these immune cells in the tumor site [43]. The natural killer (NK) cells were discovered for their ability to rapidly recognize and efficiently kill tumor cells [44], and to release a number of cytokines that regulate both innate and adaptive immune responses [45]. The infiltration of NK cells in TME was associated with improved prognosis in triple-negative breast cancer [46] and colorectal cancer [47]. Dendritic cells (DCs) are pivotal regulators of the adaptive immune response, and their potent antigen presenting ability is considered as critical factor in antitumor immunity [48]. Many studies reported that tumor-infiltrating DCs have been found in the TME in many different cancers, including lung cancer, colorectal cancer, breast cancer, head and neck cancer, bladder cancer, gastric cancer, and ovarian cancer [49]. Due to the complex phenotype and cancer heterogeneous, DC infiltration in TME has controversial results in predicting clinical outcomes in different tumors [50]. Moreover, tumor-associated neutrophils also compose a significant part of the inflammatory cell infiltrate in several tumor types [51]. In our study, we integrated the genomic profiles and deconvolution algorithm method to accurately resolve relative proportions of diverse TIICs subpopulation. CIBERSORT, which has been deemed to be the most accurate method available, not only is powerful enough to discriminate TIICs in cancer, but also could remain consistency cross different genomic data resources [52]. Using CIBERSORT, we directly compared the alteration of 22 TIICs between paired HNSC tissues and adjacent non-cancer tissues. To our knowledge, the present study was the most comprehensive analysis of the clinical impact of the TIICs in HNSC to date. We found that the proportions of naïve B cells, monocytes, resting mast cells, and CD8^+^ T cells in HNSC tissues were significantly lower than adjacent non-cancer tissues, while the proportion of activated mast cells and M0 macrophages in HNSC tissues was significantly higher than adjacent non-cancer tissues. Moreover, the proportions of some TIICs were significantly associated with clinical parameters. Taking together, our results illustrate the dichotomy (tumor-promoting and tumor-suppressing) of TIICs populations in HNSC. Therefore, different TIICs in TME could play various roles in HNSC development and progression, far more complex than anticipated.

To complement our gene-centric survival analysis, we conducted an analysis of prognostic associations for 22 immune subpopulations in HNSC. We found that Tregs was associated with improved outcome, while M0 macrophages was associated with poor outcome. Regulatory T cells (Tregs) are able to suppress antitumor immunity in some solid tumors and support the establishment of an immunosuppressive microenvironment [53–55]. Several contradicting conclusions have been drawn recently concerning the prognostic value of Tregs in cancer, in which Treg infiltration may play a positive or negative role in anti-tumor effects. At present, some studies have reported that high tumor-infiltrating Tregs were significantly associated with worse outcome in breast cancer [56], hepatocellular carcinoma [57], pancreatic ductal adenocarcinoma [58], lung cancer [59], gastric cancer [60], and ovarian cancer [61]. However, high densities of tumor-infiltrating Tregs in colorectal carcinoma are reported to be correlated with worse or better outcomes [62]. In consistent with our analysis, Lukesova et al. reported that HNSC patients with elevated Tregs levels had significantly better overall survival [63]. Due to tumor heterogeneous, we hypothesized that Tregs play different roles in different tumor types and different tumor sites in the same tumor. Macrophages are the most abundant population of TIICs in tumor microenvironments. M0 macrophages can differentiate into two further phenotypes: antitumor M1 and pro-tumor M2, under different stimulation. M0, M1, and M2 macrophages can reside simultaneously in the tumor tissues, and undifferentiated macrophages (M0) can be readily induced by cytokines and other stimuli, such as interferon-γ (IFNγ) and/or lipopolysaccharide (LPS) orient to M1 phenotype, and combination of IL4/IL13 orient to M2 macrophages, respectively [64–66]. In our analysis, we did not find an association between M1, M2 macrophages and clinical outcomes. In contrast with M1 and M2 macrophages, a higher proportion of M0 macrophages predicted poor OS and DFS in HNSC patients. We hypothesis that M0 macrophages have an impact on HNSC biology, which were influenced by some stimuli in HNSC progression, and deserve further attention in future studies.

Additionally, we identified the immune-related DEGs and found that biological processes of these DEGs mainly involved immune response, inflammatory response, and cell–cell signaling, and KEGG pathways were concentrated on cytokine–cytokine receptor interaction and hematopoietic cell lineage. Then, we screened the top 20 hub genes by Cytoscape software, and indicated that interleukin 17A (IL17A), colony-stimulating factor 2 (CSF2), interferon gamma (IFNG), cytotoxic T lymphocyte-associated protein 4 (CTLA4), forkhead/winged helix transcription factor P3 (FOXP3), CD40 ligand gene (CD40LG), CD19, and CD27 were correlated with overall survival. Although the diverse roles of above genes in tumor development had been reported, the exact mechanism in head and neck cancer is known little, which is also the focus of our future study.

However, there were some limitations in the present study. First, TCGA is a public database, clinical characteristics were not comprehensive, which might lead to potential error or bias. Second, all TCGA data were collected from Western countries, and there are some differences in the regions and human races. Third, the methodology for interpreting immune infiltration and the appropriate cut-off value needs to be standardized. Finally, our results were based on genomics data mainly of cross-sectional designs. Thus, it is necessary to further validate our findings using a longitudinal study design and/or basic cell and animal experiments.

## Conclusions

Taken together, we analyzed the 22 immune infiltrating cell subgroups in HNSC samples based on RNA sequencing data using CIBERSORT tool. In the present study, we identified some TIICs were associated with clinicopathological parameters, and determined that Tregs and M0 macrophages were independent prognostic factors in HNSC patients. Then, we identified the immune-related DEGs, predicted the functions of DEGs, and screen the prognosis-related hub genes in HNSC. However, further clinical investigation and basic experiments are needed to validate our results, and uncover the molecular mechanisms interlinking TIICs in HNSC and their roles in prognosis and therapy.

## Supplementary Material

Supplementary Figures S1 and S2

## Abbreviations

CSF2: colony-stimulating factor 2
CTLA4: cytotoxic T lymphocyte-associated protein 4
DC: dendritic cell
FOXP3: forkhead/winged helix transcription factor P3
HNC: head and neck cancer
HNSC: head and neck squamous carcinoma
HPV: human papillomavirus
IFNγ: interferon-γ
IL17A: interleukin 17A
LPS: lipopolysaccharide
NK: natural killer
TIIC: tumor immune infiltration cell
