# Supplementary material for: Profiles of immune cell infiltration in head and neck squamous carcinoma
Source: Biosci Rep. 2020 Feb 25;40(2):BSR20192724. doi: 10.1042/BSR20192724 (PMC7042147; doi:10.1042/BSR20192724)
Supplement: Supplementary Figures S1 and S2 [file BSR-2019-2724_supp.pdf]

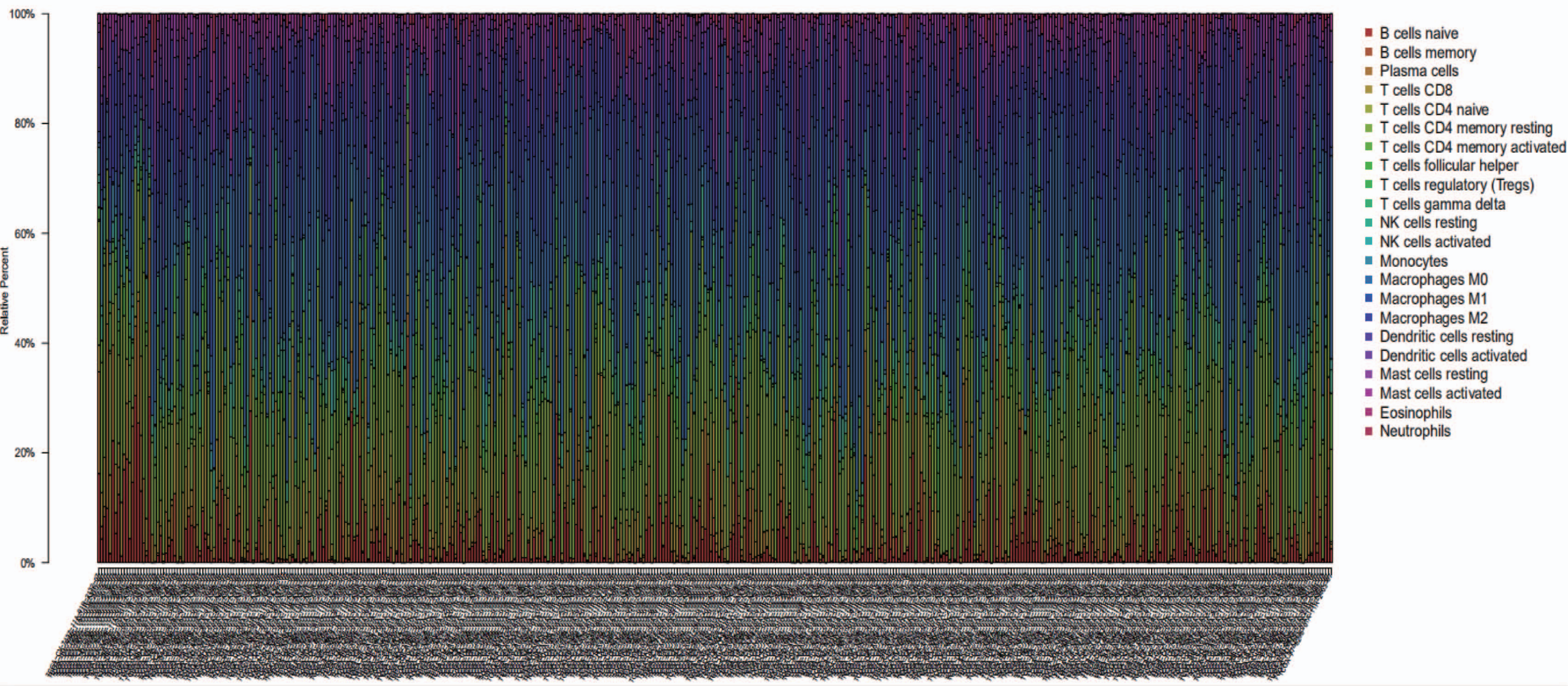

100%
80%
60%
40%
20%
0%
Relative Percent
B cells naive
B cells memory
Plasma cells
T cells CD8
T cells CD4 naive
T cells CD4 memory resting
T cells CD4 memory activated
T cells follicular helper
T cells regulatory (Tregs)
T cells gamma delta
NK cells resting
NK cells activated
Monocytes
Macrophages M0
Macrophages M1
Macrophages M2
Dendritic cells resting
Dendritic cells activated
Mast cells resting
Mast cells activated
Eosinophils
Neutrophils

Figure S1. The relative percent of 22 TIICs in HNSC samples

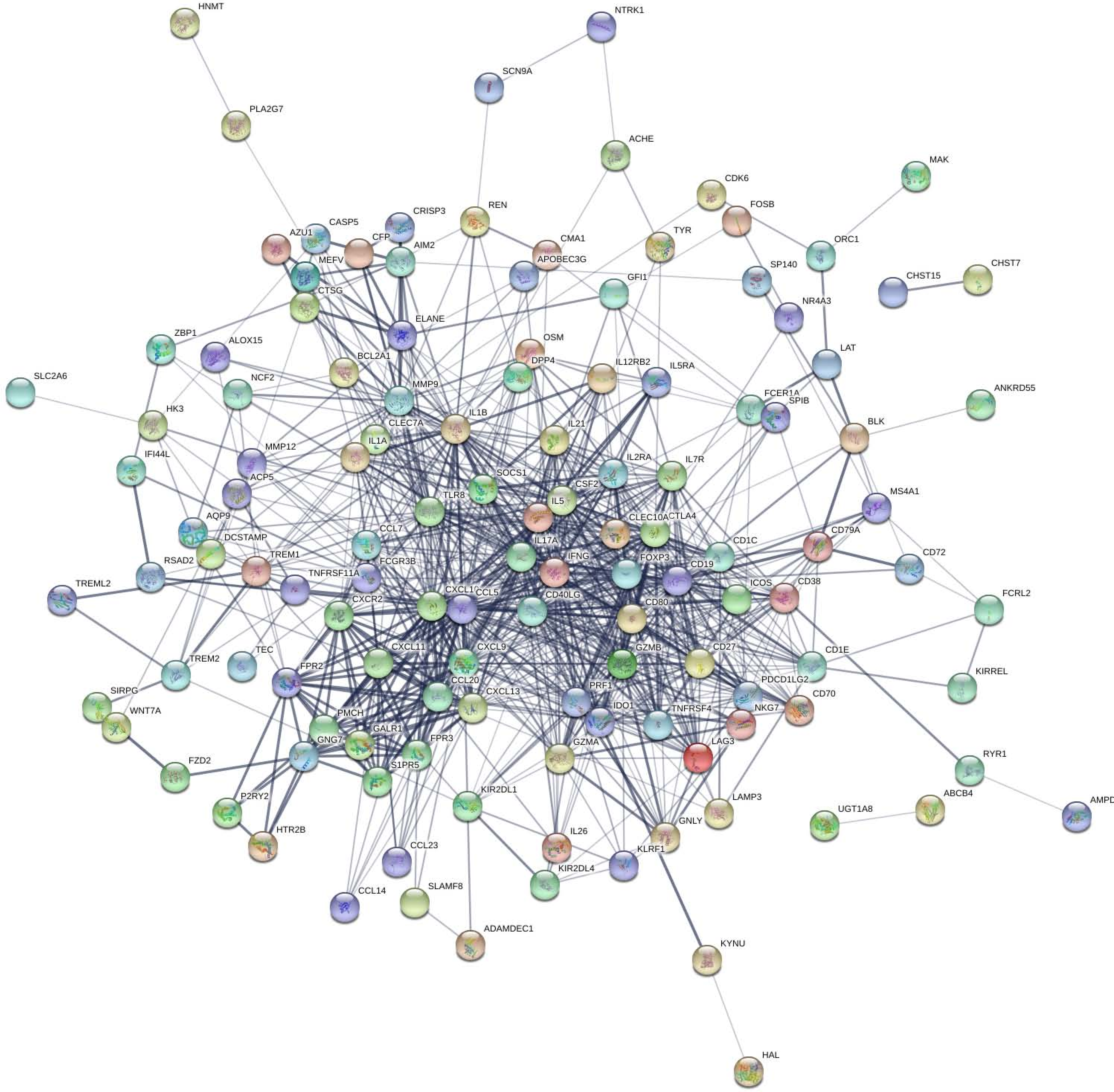
HNMT
NTRK1
SCN9A
PLA2G7
ACHE
MAK
CDK6
REN
FOSB
CRISP3
CASP5
AZU1
CFP
AIM2
CMA1
TYR
ORC1
MEFV
APOBEC3G
CHST15
CHST7
SP140
GFI1
CTSG
NR4A3
ELANE
ZBP1
ALOX15
OSM
LAT
BCL2A1
DPP4
IL12RB2
IL5RA
SLC2A6
NCF2
MMP9
ANKRD55
FCER1A
SPIB
HK3
IL1B
CLEC7A
BLK
IL1A
IL21
MMP12
IFI44L
IL2RA
IL7R
ACP5
SOCS1
CSF2
MS4A1
TLR8
IL5
AQP9
CLEC10A
CTLA4
CD79A
DCSTAMP
CCL7
IL17A
CD1C
CD72
RSAD2
TREM1
IFNG
FOXP3
FCGR3B
CD19
TREML2
TNFRSF11A
ICOS
CD38
CXCL10
CCL5
FCRL2
CXCR2
CD40LG
CD80
TEC
CXCL11
CXCL9
GZMB
CD27
CD1E
TREM2
FPR2
KIRREL
SIRPG
PDCD1LG2
CCL20
CXCL13
PRF1
CD70
WNT7A
PMCH
IDO1
TNFRSF4
NKG7
GALR1
GNG7
FPR3
GZMA
LAG3
FZD2
RYR1
S1PR5
P2RY2
KIR2DL1
LAMP3
ABCB4
UGT1A8
AMPD
HTR2B
GNLY
IL26
CCL23
KLRF1
KIR2DL4
SLAMF8
CCL14
ADAMDEC1
KYNU
HAL

Figure S2. PPI network of common DEGs in STRING database. The minimum required interaction score was set as 0.4.
